# Renal Assessment in Acute Cardiorenal Syndrome

**DOI:** 10.3390/biom13020239

**Published:** 2023-01-27

**Authors:** Piotr Łagosz, Jan Biegus, Szymon Urban, Robert Zymliński

**Affiliations:** 1 Institute of Heart Diseases, Wroclaw Medical University, 50-556 Wroclaw, Poland; 2 Institute of Heart Diseases, University Clinical Hospital, 50-556 Wroclaw, Poland

**Keywords:** heart failure, cardiorenal syndrome, kidney injury, renal function, renal biomarkers

## Abstract

Cardiorenal syndrome (CRS) is a complex, heterogeneous spectrum of symptoms that has kept cardiologists awake for decades. The heart failure (HF) population being burdened with multimorbidity poses diagnostic and therapeutic challenges even for experienced clinicians. Adding deteriorated renal function to the equation, which is one of the strongest predictors of adverse outcome, we measure ourselves against possibly the biggest problem in modern cardiology. With the rapid development of new renal assessment methods, we can treat CRS more effectively than ever. The presented review focuses on explaining the pathophysiology, recent advances and current practices of monitoring renal function in patients with acute CRS. Understanding the dynamic interaction between the heart and the kidney may improve patient care and support the selection of an effective and nephroprotective treatment strategy.

## 1. Introduction

The interaction between the heart and the kidney is governed by a network of numerous complex and multi-level feedback loops. In recent years, great emphasis has been placed on understanding the spectrum of symptoms called cardiorenal syndrome (CRS) and the pathophysiological phenomena involved, in which failure of either the heart or the kidney results in the injury to the other. In 2010, CRS was classified into five subtypes by Ronco, based on acute or chronic affection and whereby a disorder of the heart induced renal dysfunction or contrariwise [1] (Table 1). The purpose of this review is to present the pathophysiology and clinical significance of acute CRS with an overview and comparison of novel methods of monitoring renal function.

The classic understanding of CRS was based on the belief that the kidneys are stimulated by lowered renal plasma flow to retain water and sodium in order to improve arterial filling and maintain adequate perfusion to vital organs. Indeed, the basic function of the heart, i.e., providing sufficient blood in- and outflow, is significantly impaired in conditions of compromised systolic and diastolic function of the heart [2].

Initially, it leads to the activation of various hemodynamic and neurohormonal compensatory mechanisms [3,4]. Reduced cardiac output and prerenal hypoperfusion stimulates the sympathetic nervous system (SNS), the renin–angiotensin–aldosterone system (RAAS) and non-osmotic release of vasopressin increasing preload as a consequence of fluid and salt retention [5,6,7]. In addition to the vasoconstrictive response mediated by the SNS, as a result of stimulation of the juxtaglomerular complex in the nephron, the release of renin is increased. This leads to further activation of the RAAS, with a synergistic effect of angiotensin and aldosterone on systemic vascular resistance and sodium reabsorption [8]. In a recent study on 211 patients with acute heart failure, RAAS overactivity was linked to worse renal function, diuretic and natriuretic response and poor outcomes [9]. Increased vasopressin levels result in fluid reabsorption and excretion of more concentrated urine. Moreover, inappropriate stimulation of the RAAS axis results in the formation of reactive oxygen species (ROS), caused by activation of nicotinamide adenine dinucleotide phosphate by angiotensin II. In the setting of developing kidney injury, the homeostasis between nitric oxide (NO) and ROS is shifted toward oxidative stress [10]. Physiologically, NO represents a significant role in paracrine and autocrine signaling as a vasodilatative, natriuretic and vascular endothelial growth factor, as well as an inhibitor of smooth muscle cell proliferation, atherogenesis and platelet aggregation [11]. Disproportionate ROS production accelerates atherosclerosis, which is the most common factor leading to the development of heart failure (HF). A combination of these factors contributes to heart and renal tubular cell fibrosis in the long term.

One of the main determinants of renal dysfunction in CRS is elevated venous pressure [12]. An analysis of studies involving hemodynamic measurements provided evidence for the association of elevated right atrial pressure (RAP) and central venous pressure with deterioration of renal function [13,14].

**Figure 1:**
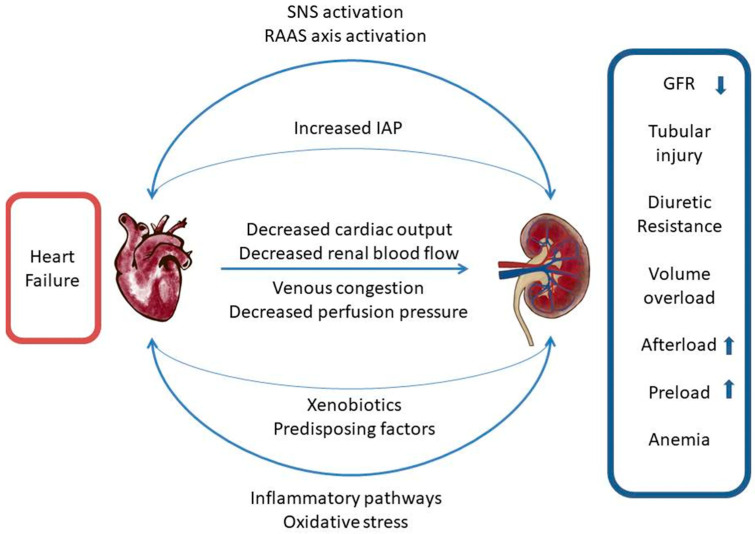
Cardiorenal network of interactions. SNS—sympathetic nervous system, RAAS—renin-angiotensin-aldosterone system, IAP—intraabdominal pressure, GFR—glomerular filtration rate.

In patients who presented with acute HF, fluid overload manifesting as pulmonary and systemic congestion is the most prevalent concern, causing effort intolerance and edema [15]. Accompanied by elevated central venous pressure, this ominous condition results in renal venous hypertension, increased renal resistance and decreased renal blood flow [16,17]. In the early phase, efferent arteriolar constriction leads to elevated intraglomerular pressure and therefore preserves the filtration fraction. Only after compensatory capacity is depleted, a decrease in glomerular filtration rate (GFR) can be observed. Increased proximal tubular sodium and water reabsorption eventually results in oliguria and worsening congestion. Recently, an experimental, invasive treatment method based on lowering inferior vena cava pressure through a specially designed Doray catheter has emerged. The first in-human study proved the safety of the method and initial results showed an improved diuretic response, opening the way for further research on its efficacy [18]. In similar fashion, elevated intra-abdominal pressures (IAP) observed in HF may contribute to renal dysfunction by reducing the renal filtration gradient and shunting blood from the kidney, thus starting glomerular necrosis, tubular injury and progressing towards renal insufficiency [19] (Figure 1). Since the population with HF is characterized by advanced age and multimorbidity, as well as that chronic kidney disease (CKD) and HF share similar risk factors, patients newly diagnosed with HF are frequently burdened with CKD. Literature data show that CKD has been observed in more than half of chronic HF patients and is an independent risk factor of mortality [20,21]. Often, it is impossible to determine which condition occurred first.

## 2. Monitoring of the Renal Function

The current European Society of Cardiology (ESC) guidelines recommend only creatinine, urea and natriuresis assessment. There are a number of biomarkers studied for their utility in the evaluation of glomerular and, conventionally omitted from the worsening of renal function (WRF) definition, tubular function. Given the heterogeneous nature of HF (e.g., de novo HF vs. acute decompensated HF (ADHF)), different biomarkers could potentially be applicable in specific clinical situations [22]. Noteworthy, novel, artificial intelligence-based approaches to analyze the nature of WRF in HF have emerged. Clustering, a machine-learning technique that distinguishes smaller subgroups in studied populations, revealed different HF patients phenotypes regarding WRF risk and characteristics [23,24,25].

### 2.1. Glomerular Filtration

There are numerous definitions of acute kidney injury (Table 2). The traditional approach to monitoring kidney function in HF focused on serial assessments of creatinine levels and estimated glomerular filtration rate (eGFR) calculations. Elevated serum creatinine requires careful evaluation, since it depends on the clinical context and does not always indicate WRF. Creatinine dynamics are characterized by high inertia, meaning that its plasma levels increase when kidney injury is already established. In acute conditions, it can lead to incorrect therapeutic decisions and cessation of therapy [26,27]. Aggressive diuretic treatment, used in ADHF presenting with congestion, can naturally lead to increased creatinine levels by reducing the volume of intravascular fluid and increasing hemoconcentration. Importantly, WRF occurring during decongestive therapy, with achieved target diuresis, natriuresis and clinical improvement is related to a better outcome [28]. This phenomenon has been called pseudo-WRF [29,30]. Moreover, the treatment used for RAAS inhibition may in fact lower baseline glomerular filtration rate (GFR), yet improve long term outcome. In this case, which is particularly true for HF with reduced ejection fraction, benefits outweigh risks of WRF and do not reflect renal injury [31].

The gold standard for measuring GFR is using plasma or urinary clearance of an exogenous filtration marker. Traditionally, iothalamate or inulin is used for this purpose, since no renal absorption and secretion occurs [32]. It is a complex procedure that is not routinely performed in everyday clinical practice. Therefore, formulas have been derived to estimate GFR using endogenous molecules and variables such as age, weight, gender and race. A comparative study implicated that none of the creatinine-based methods were accurate in calculating eGFR in HF patients [33]. Depending on patients’ profiles, specific methods of estimating GFR have their advantages and drawbacks. Among commonly used equations, the Chronic Kidney Disease Epidemiology Collaboration (CKD-EPI) formula was proven to be the most accurate in predicting GFR in chronic heart failure, while the Cockcroft–Gault formula has been highlighted to surpass the simplified Modification of Diet in Renal Disease (sMDRD) formula’s ability to predict outcome in heart failure [34,35]. Still, despite the limitations, estimations based on serum creatinine are the most frequently incorporated in clinical practice. Plasma creatinine is a breakdown product of creatine phosphate from muscle and protein metabolism; however, it is subject to error due to dynamic secretion by the renal tubules [36]. Endogenous filtration marker cystatin C (CysC) has now been proposed as a substitute to overcome this issue [37]. CysC is a molecule that functions as a cysteine proteinase inhibitor and is synthetized by nucleated cells at a constant rate. Unlike creatinine, it is freely filtered by the glomeruli and broken down in the process of reabsorption, so its blood level correlates with the glomerular filtration rate. An additional advantage of this measurement is that it is independent of gender, weight, height, muscle mass and age [38]. Nonetheless, both markers can be misinterpreted because their concentrations depend on the individual clinical characteristics of patients. In patients with heart failure, obesity or cachexia may be common factors encountered. Reduced lean muscle mass may lower creatinine levels and lead to overestimation of GFR, while excessive body weight, inflammation and thyroid dysfunction can lead to an increase in cystatin C and underestimation of GFR. [39,40,41]. Creatinine concentration can also vary with diet, particularly protein supply [42]. Given this, assessment should never be made in isolation from the patient’s clinical status. Studies in HF population indicate that CysC might be an accurate tool for prognosis of outcome [43,44].

Another problem of numerous GFR-calculation formulas is their impact on optimizing drug doses. Depending on the study, different GFR cut-off points for safe drug use have been established based on various formulas. In the patients with HF, we most often encounter the problem of existing indications for the use of novel oral anticoagulants. During clinical trials, GFR was mostly estimated based on the Cockcroft–Gault formula, in the setting of stable patients without dynamic changes in renal function. Therefore, eGFR as the sole determinant of renal function is sometimes misleading and requires a broader perspective.

A method that can provide a more accurate assessment of kidney function is the evaluation of renal functional reserve. It is defined as the kidney’s ability to increase glomerular filtration of residual nephrons to maintain GFR under conditions of nephron damage [45]. Physiologically, the kidney can maintain GFR with only 50% of functioning nephrons. Therefore, it can be a precise way to predict the chances for renal recovery in AKI and may provide early detection of renal injury before changes in GFR. There is growing evidence for utility of RFR in risk stratification of developing chronic kidney disease [46]. Yet, the feasibility of this test is limited because it requires complicated protein loading protocols and may be hard to conduct in acute conditions.

### 2.2. Urine Composition and Biomarkers of Glomerular Integrity

Recently, the focus has been placed on urine composition testing as a material that is simple to collect. The urine analysis provides rapid information on renal function and lacks the inertia that creatinine determinations have. Assessment of electrolyte composition, specifically sodium concentration, is recommended by the current ESC guidelines [47]. It has been proven that spot urinary sodium is not correlated with eGFR, which confirms that it may carry different information that may improve patient profiling [48]. Determination of spot urinary sodium two hours after starting decongestive treatment is recommended to assess the diuretic response [49]. The urinary sodium level in ADHF seems to be the most powerful prognostic tool at admission and during the first days of decongestion [50]. This parameter has gained special importance with reports of better outcome in patients with satisfactory diuretic response and optimal decongestion [51,52,53].

Albuminuria is a simple parameter to assess podocyte integrity and has been used for years in nephrology [54]. When the glomerular membrane becomes damaged, its permeability to large molecules, including various protein fractions, increases [55]. As a result, we can observe a rise in urinary albumin, a parameter conventionally used for monitoring the onset of chronic kidney disease in diabetic patients. Since patients with HF are a group frequently burdened with diabetes or hypertensive nephropathy, microalbuminuria is highly prevalent [56]. It was found to be a powerful independent marker of adverse outcome in chronic HF [57,58].

Urine sediment, being a proven method of diagnosing kidney injury, still remains unexplored in the setting of HF [59]. However, a small study has shown that this simple test can be a helpful tool in predicting AKI [60].

Galectin-3 (gal-3) is a pleiotropic member of the carbohydrate-binding proteins family known as lectins. It exhibits antimicrobial and antifungal activity and takes part in cell adhesion, chemoattraction and cytophysiological processes [61]. It promotes fibrosis, leads to myocardial remodeling and may contribute to heart and kidney failure. Its exact role in the development of HF is still being explored, but several studies have emerged that suggest a link between the aggravated myocardial fibrosis and Gal-3 and stated that it may be a future target for pharmacotherapy [62,63]. The profibrotic properties of gal-3 may be one of the main factors leading to kidney damage [64]. In a study of gal-3 inhibitors conducted on rats with induced hypertension and HF, the efficacy of gal-3 inhibitors in reducing proteinuria and improving their function was proven, resulting in improved renal function [65]. Concerning humans, in a small observation made on 260 patients with chronic heart failure, Gal-3 was linked to 1-year progression of renal dysfunction [66]. Proenkephalin A is an endogenous opioid polypeptide hormone with a cardiodepressive effect, which acts primarily on delta opioid receptors. It is suggested to have a regulatory role in diuresis and natriuresis. A study including 1908 patients indicated its prognostic value for worsening renal function and both in-hospital and 1-year mortality in patients hospitalized for ADHF [67].

### 2.3. Biomarkers of Tubular Injury

**Figure 2:**
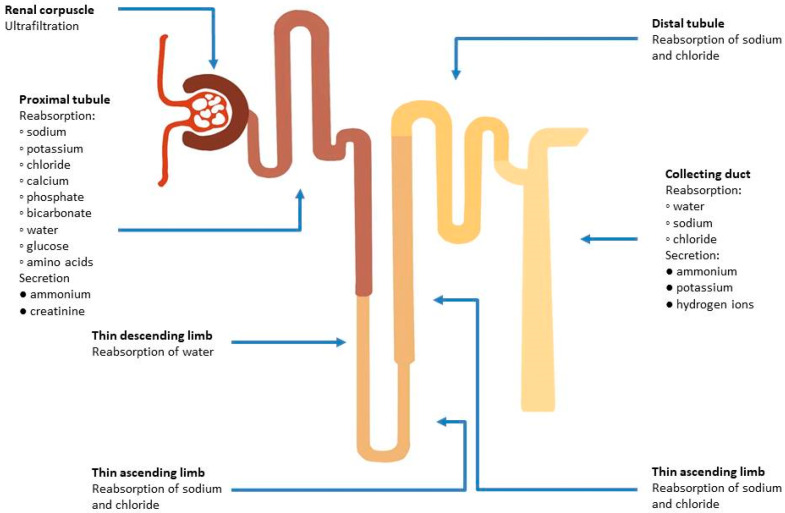
Functions of different parts of the nephron in maintaining fluid and electrolyte balance.

Considering the complex characteristics of renal tubules, it is a challenge to assess their function (Figure 2). This results in a large number of studied biomarkers, which allow early detection of AKI and provide predictive and prognostic value. The assessment of renal tubular function can be a useful tool in differentiating between true and pseudo WRF [68]. Yet, there is no consensus on evaluation of tubular function, and its utility and some assays are used only for research purposes, since they are not available for clinical use.

**Figure 3:**
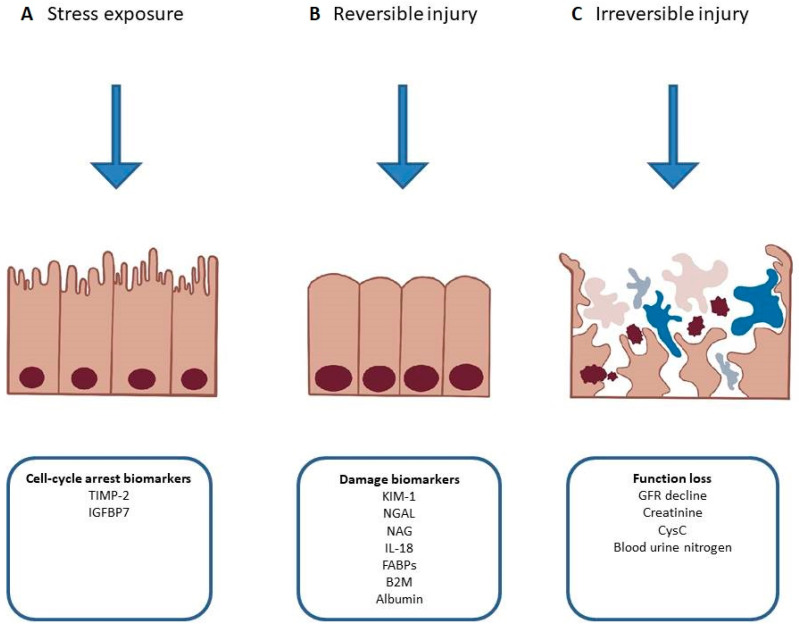
Phases of acute kidney injury and relevant biomarkers. (**A**) Release of cell-cycle arrest biomarkers in response to exposure to non-damaging stimuli. (**B**) Potentially reversible injury resulting in changed metabolism or expression of damage biomarkers. (**C**) Persistent injury resulting in necrosis, apoptosis and kidney dysfunction. TIMP-2—tissue inhibitor of metalloproteinase 2, IGFBP7—insulin-like growth factor-binding protein 7, KIM-1—kidney injury molecule 1, NGAL—neutrophil gelatinase-associated lipocalin, NAG—*N*-acetyl-ß-d-glucosaminidase, IL-18—interleukin-18, FABPs—fatty acid-binding proteins, B2M—beta-2 microglobulin, GFR—glomerular filtration rate, CysC—cystatin C.

Neutrophil gelatinase-associated lipocalin (NGAL) is a molecule involved in innate immunity. The primary function of NGAL is in sequestering bacterial siderophores, thus limiting iron usage by bacteria and slowing their growth. It is secreted by neutrophils during bacterial infections and to some extent by organs including the kidney, prostate, gastrointestinal and respiratory tracts [69]. It can be found both in serum and urine and it increases in AKI [70]. There are biased data on the prognostic value of NGAL. Previous observations made on enrolled HF patients suggested an association with WRF and adverse outcome [71,72,73,74,75,76]; however, the largest, multicenter cohort study to date does not support the routine use of serum NGAL for early detection of WRF in AHF treated with diuretic agents. According to the AKINESIS trial, serum NGAL has not been shown to be superior to creatinine in predicting adverse in-hospital outcomes and WRF [77]. On the other hand, studies have implicated that tubular damage might occur in the absence of an increase in serum creatinine, opening up a possibility of early detection of renal injury and timelier deployment of therapy, suggesting that the definition of AKI should be re-assessed [78,79] (Figure 3). A study on urinary NGAL has shown that it can identify high-risk ADHF patients, and predict WRF and early outpatient mortality. Moreover, it has been reported that in a longer, 1-year follow-up period, increased urinary NGAL may predict the risk of HF rehospitalization [80]. It should be noted that NGAL may be overexpressed in conditions of hypertension, hypoxemia, infections, anemia and malignancies, which are greatly prevalent among patients with HF [81,82,83,84,85].

Kidney Injury Molecule 1 (KIM-1) is a biomarker expressed by the nephron in response to ischemic kidney injury [86]. Urinary KIM-1 has been proven to be a sensitive and specific marker for early AKI and may be useful in differentiating between acute tubular necrosis and other forms of renal injury [87]. Some studies suggest that elevated uKIM1 can indicate tubular damage in states of both acute and chronic heart failure and may be utilized in the prediction of CRS [88,89]. Data on the prognostic value of serum KIM-1 in HF are scarce; however, the study designed for investigation of KIM-1 utility in both chronic and acute HF showed that serum KIM-1 is only moderately associated with clinical outcome [90]. Authors of the Ascend-HF study suggest that pathophysiologic process involved in ADHF, i.e., venous congestion and decreased cardiac output, may affect glomerular filtration and tubular injury and may not be as severe as previously thought [91]. Conclusions from smaller study focused on possible biomarker utility in CRS, stated that uKIM-1 levels were strongly correlated with congestion, serum NT-proBNP, left ventricle dysfunction and NYHA stage and in a 1-year follow-up, KIM-1 was associated with lower overall survival in chronic heart failure [92].

In the same study, similar observations were made for other biomarker, *N*-acetyl-ß-d-glucosaminidase (NAG) [92]. NAG is a high molecular weight biomarker which originates for proximal tubular cells and serves as a lysosomal enzyme. In addition to the aforementioned study, it is suggested to be significant predictor of all-cause mortality in chronic HF based on 10-year follow-up [93].

Interleukin-18 (IL-18), known as interferon-gamma inducing factor, is a proinflammatory cytokine produced by both hematopoietic and non-hematopoietic cells, but is mainly associated with macrophages [94]. In the kidney injury, it is released from the proximal tubule [95]. A prospective, multicenter study in the setting of ADHF showed that elevated urinary IL-18 was associated with a 3.6-fold risk of AKI compared with lower levels in the adjusted analysis [96].

Angiotensinogen, which belongs to the family of serpin proteins, is a globulin synthesized in the liver. In study mentioned above, it presented the strongest predictor of the progression of AKI and worsening of AKI with death in ADHF patients [96].

Uromodulin or Tamm–Horsfall protein is a kidney specific protein produced by cells of the thick ascending limbs and early distal convoluted tubules, which is found in urine in polymerized and non-polymerized forms under normal circumstances. So far its several important functions have been identified including ion transportation in the ascending limb in nephrons, fluid and electrolyte balance, prevention of kidney stones and innate immunity of the urinary tract [97]. Moreover, mutations in the uromodulin gene (UMOD) cause autosomal dominant tubulointerstitial kidney disease or medullary cystic kidney disease and some variants are associated with the development of hypertension and chronic kidney disease, which proves its important role in the correct development and functioning of the kidney [98,99,100]. LaFavers’ review paper takes a look at new findings on physiological concepts surrounding uromodulin, the separate regulatory pathways for its different forms, and it provides recommendations for reporting uromodulin levels [101]. A study conducted by M. Pruijm et al. confirmed the utility of uromodulin as a biomarker of tubular function, and large study on more than 3000 patients undergoing coronary angiography further proved its value in prognosis of all-cause mortality [102,103]. Another novel biomarker alpha-1 microglobulin (A1M) is a small globular protein synthesized by the liver, which is typically entirely reabsorbed by the renal tubule. In vivo, it has an antioxidant function [104]. The concentration of both of the molecules was observed to rise in the presence of tubular cell damage. Independently from GFR, both low levels of urine uromodulin and high levels of urine A1M may be associated with a greater risk of developing cardiovascular disease and mortality [105].

Tissue inhibitor of metalloproteinases 2 (TIMP-2) and insulin-like growth factor-binding protein 7 (IGFBP7) are cellular stress biomarkers, involved in the cell cycle arrest in the G1 phase. By inhibition of cell division, they prevent the multiplication of damaged DNA in the case of cellular injury. Both are secreted by renal tubules—TIMP2 mainly by distal tubule cells, while IGFBP7 is primarily secreted by proximal tubule cells [106]. TIMP-2 and IGFBP7 activity is increased in harmful conditions such as ischemia, oxidative stress, inflammation and others. Noteworthy, the TIMP2/IGFBP7 ratio is a part of the commercially available NephroCheck Test. In a retrospective analysis, it was shown that it can predict the occurrence of AKI and is more reliable compared to CysC, NGAL, KIM-1, liver fatty acid-binding protein and IL-18. The multicenter, randomized controlled trial has proven that monitoring with these markers and appropriate treatment management can lead to a reduction in AKI [107].

Fatty acid-binding proteins (FABPs) are a family taking part in fatty acid transport and metabolism. With ongoing research, more proteins belonging to this family are being discovered. In this review, two site-specific FABPs will be discussed. Heart FABP, also called FABP-3, mainly presents inside cardiomyocytes, skeletal muscles and kidney together with liver FABP, known under the name of FABP-1, located in the hepatocytes and intestines. Urinary levels of each of them have been proven to rise with ischemic tubular injury and have been associated with an increased risk of AKI development [108,109,110]. In addition, the results of a study highlight the association between elevated heart FABP and the risk of adverse outcome in patients with chronic heart failure [108].

Beta-2 microglobulin (B2M) is a component of major histocompatibility complex class I molecules encoded by the B2M gene and secreted by nucleated cells at a constant rate. Urinary levels of B2M rise in tubular injury due to reduced reabsorption in the proximal tubule. Results from study on rat models showed an early increase in B2M in AKI [111]. Moreover, it is a marker independent of muscle mass, sex, age and race. In comparison to eGFR it appears to have less specificity and better sensitivity for prognosis of deteriorated renal function at one year [112]. Combined evidence presented in a meta-analysis suggested that B2M may be also linked to an increased risk of cardiovascular disease [113].

An overview of characteristics, strengths and limitations of selected tubular injury biomarkers is provided in Table 3.

### 2.4. Biomarkers of Endothelial Dysfunction

Endothelial dysfunction remains in the spotlight because of its central role in the pathogenesis of cardiovascular and renal diseases and the crosstalk between the heart and the kidney. It is one of the important contributors in the development of CRS, not only leading to the progression of atherosclerotic lesions, but also involved in kidney injury [114]. Various markers of endothelial dysfunction have been identified over the years, including endothelin-1, matrix metalloproteinases, tissue inhibitor of metalloproteinases, angiopoietin-like 2, endoglin, homocysteine or endothelial cell microparticles [115]. While most of them still possess numerous limitations that hinder clinical application, syndecan-1 is a biomarker of endothelial dysfunction that has found particular use in assessing renal function. It is a transmembrane proteoglycan, which reflects endothelial glycocalyx damage. In vivo glycocalyx is an ultra-thin carbohydrate-rich structure which provides protection to the vascular endothelium due to its anticoagulant and antiadhesive properties. In a pioneer study in ADHF patients, the concentration of syndecan-1 at admission was predictive for in-hospital AKI and mortality and was strongly associated with 6-month mortality [116].

### 2.5. Renal Imaging

We should keep in mind that renal imaging techniques bring a spectrum of possibilities in exploring pathophysiological pathways, and structural and functional alterations, as its capabilities continue to expand. Renal imaging complements laboratory data and may provide early diagnosis of WRF and prognostic information. (Table 4).

#### 2.5.1. Ultrasonography

Renal ultrasonography is a routine examination used in nephrology. The ability to distinguish between the fibrosis and the inflammation and to differentiate between underlying pathophysiologic processes in a standard ultrasound is limited [117]. In addition to the standard structural assessment, Doppler ultrasonography can be used to quantify renal blood flow and hemodynamics. Intrarenal venous Doppler shows the waveform of blood flow in the interlobal veins of the kidney. It is dependent on the RAP and IAP. As the RAP increases, the pattern of the Doppler spectrum changes. In physiological conditions, the waveform is continuous, while in the case of venous congestion, the flow changes to pulsatile and the spectrum splits into biphasic, in which the systolic and diastolic phases can be distinguished. With a further increase in RAP, only monophasic, diastolic flow is observed. Association with RAP was confirmed in patients with HF who underwent right heart catheterization [118]. The abovementioned patterns may be used to evaluate response to diuretic therapy and guide decongestion. An observation made in the comparison study of patients with HF and healthy subjects indicated that venous impedance index, calculated based on intrarenal venous Doppler, was significantly increased in HF and could be restored to normal after loop diuretic administration [119]. Doppler-enhanced venous flow studies reflect renal congestion and might bring important prognostic information and optimize decongestion strategies in patients with chronic HF [120]. The utility of such measurements has not yet been confirmed in large studies, but it is currently a topic of interest to researchers [121,122]. Assessment of the renal resistive index can provide prognostic information in various scenarios. In animal models, contrast-enhanced ultrasonography was proven to be a valuable tool for evaluation of renal perfusion and microvascular function [123]. In the near future, this method may be implemented in daily practice to diagnose and monitor AKI.

#### 2.5.2. Computed Tomography (CT)

CT is a method that allows structural and functional evaluation of the kidney, but has some limitations that preclude its use in serial evaluation. Currently, it is not feasible for serial assessment of the kidney in patients with renal failure due to the risk of inducing post-contrast nephropathy and exposure to high doses of radiation [124].

#### 2.5.3. Magnetic Resonance Imaging (MRI)

MRI compared to CT is an examination that avoids radiation exposure; however, complications associated with gadolinium-based contrast and lower availability should be taken into account [125]. Imaging with this technique uses a strong magnetic field, which can be a relative contraindication for selected HF patients, for instance, those who have a non MRI-friendly cardioverter-defibrillator implanted. MRI may provide various microstructural and functional information about the kidney, depending on the used sequence. In recent years, the literature on the advances in the use of MRI for renal assessment is rapidly expanding. The progress that has been made resulted in the formulation of general principles for the acquisition, processing, and analysis of data to promote standardization and facilitate clinical implementation of further explained techniques [126,127,128,129,130].

T1 and T2 mapping, diffusion-weighted imaging (DWI) and diffusion tensor imaging (DTI) are sequences used for the evaluation of fibrotic alterations in the kidney. To date, only a few pioneer studies in animals and humans pointed out that functional MRI could be a prognostic tool for AKI and progression of CKD [131,132,133]. This subject still requires further research.

Arterial spin labeling (ASL) is a technique that uses labeled water tracing for regional perfusion mapping. Although it is mostly restricted to research settings, the latest studies have stated that cortical perfusion decreases in CKD proportionally to stages of CKD and correlates to eGFR [134].

Blood oxygen level dependent (BOLD) imaging is a technique, which relies on paramagnetic properties of deoxyhemoglobin and T2-weighted sequence. BOLD allows identification of renal hypoxia and requires no contrast. It remains a diagnostic tool used mainly in scientific research due to difficulties in analyzing real-time estimated oxygen delivery and consumption. The relation between renal T2* and renal tissue pO2, using gold-standard invasive physiological measurements, has been proven to be highly correlated in an animal model [135]. Increasing experimental and clinical data indicate that reduced renal oxygenation and mitochondrial dysfunction are significant contributors to AKI development and CKD progression [136,137]. Moreover, the latest studies showed that monitoring urine oxygenation may be a useful predictor for AKI development in septic and cardiac surgery patients [138,139]. Hence, it is imperative to study the pathophysiology behind this phenomenon further, identify biomarkers of renal hypoxia and improve techniques that will allow for accurate assessment of renal oxygenation. Novel therapies to restore the integrity of oxygen delivery and consumption may improve clinical outcomes. Currently, the activation of hypoxia-inducible factor-1α has been demonstrated to have a beneficial effect on renal function and metabolism [140].

#### 2.5.4. Nuclear Imaging

Renal nuclear imaging is a frequently used modality for renal assessment. Most commonly, it is based on ^99m^technetium bound to non-metabolized molecules with known pharmacokinetics; in this case, dimethylenetriaminepentaacetic (DTPA) and acidmercaptoacetyltriglycine (MAG3) are the tracers of choice for evaluation of GFR and RBF. This method has good availability and reproducibility; however, it is currently not applicable in monitoring renal function due to high radiation doses and poor imaging characteristics [141].

Positron emission tomography (PET) is characterized by higher resolution compared to conventional scintigraphy, and combined with CT may provide additional data on kidney composition and function. At this time, nuclear imaging of the kidney is used mainly for research purposes.

## 3. Intra-Abdominal Pressure

**Figure 4:**
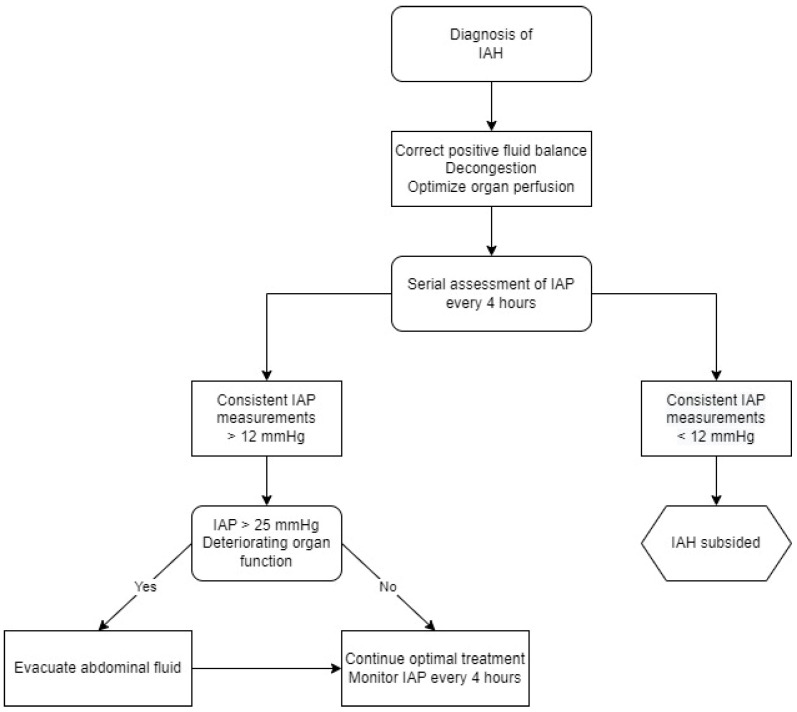
Management algorithm for intra-abdominal hypertension. IAP—intra-abdominal pressure, IAH—intra-abdominal hypertension.

Increased IAP has received scientific attention since the 19th century [142]. Not long after this phenomenon was described, the first reports appeared regarding its adverse effects on organ function. The accumulation of evidence throughout the last decades has led to a deeper understanding of the underlying pathophysiology and its impact on homeostasis. In 2004, the World Society for Abdominal Compartment Syndrome (WSACS) was established to provide unified definitions, promote research in this field and improve management. According to the created guidelines, the normal IAP in critically ill adults oscillates between 5 mmHg to 7mmHg; intra-abdominal hypertension (IAH) is defined as a sustained or repeated rise in IAP above 12 mmHg. The most ominous condition associated with elevated IAP is an abdominal compartment syndrome, which can be diagnosed with sustained IAP range above 20 mmHg, accompanied by newly diagnosed organ dysfunction or failure [143]. Because of the possible negative impact on every system in the human body and the risk of developing multiple organ failure, such patients are at the highest risk of poor outcomes [144,145]. It has been proven that this phenomenon is widespread in the population of patients hospitalized in intensive care units and represents an independent predictor for mortality and prolonged stay in the hospital. Considering the fact that the kidneys are the most vulnerable to IAP fluctuations, their evaluation is a valuable tool for monitoring in CRS. The WSACS recommends recording IAP by transurethral measurement using a Foley catheter, which is low cost, simple and reliable. This method has been validated in the setting of ADHF [146]. Elevated IAP leads to reduced renal blood flow, renal venous congestion and parenchymal compression, increased pressure in the proximal tubule and reduced renal filtration gradient [11]. As a result, the RAAS is chronically activated and a large decrease in urine sodium concentration may be observed, which contributes to the development of diuretic resistance and further disturbs the fluid balance [49,147]. The effect of IAH highly depends on its severity. With values above 15 mmHg, reduced renal filtration pressure leads to oliguria, while anuria is observed at a pressure of 30 mmHg. Furthermore, an IAP of 20 mmHg causes an increase in renal vascular resistance by 500%, shunting blood from the kidney and resulting in glomerular necrosis and tubular damage [148]. In the population admitted for management of ADHF, a high prevalence of elevated IAP was found, which was strongly correlated with impairment of renal function [149]. In the case of diagnosed IAH, it is recommended to implement an appropriate treatment, perform serial measurements every 4 h and keep a close observation for the occurrence of abdominal compartment syndrome (Figure 4).

## 4. Conclusions

The close physiological relationship of the heart to the kidney puts each HF patient at risk of developing renal failure. The proper phenotyping of patients with cardio-renal syndrome, due to their multimorbidity and heterogeneity, requires an individual approach that often goes beyond guidelines. The presented methods of diagnosing and monitoring renal function provide additional information on the actual state of the kidneys, allowing early detection, characterization of the cause and course of WRF. With the emergence of new studies on novel biomarkers of prognostic potential and the introduction of multimarker testing, we also have the opportunity for better risk stratification and timed interventions. New approaches to renal assessment will certainly be incorporated into management strategies in the near future to improve CRS care.

## Figures and Tables

**Table 1:** Classification of cardiorenal syndrome.

Classification	Trajectory	Clinical Context
CRS type I Acute cardiorenal syndrome	Acute dysfunction of the heart leading to acute kidney injury	ADHF Cardiogenic shock Acute valvular insufficiency
CRS type II Chronic cardiorenal syndrome	Chronic dysfunction of the heart leading to progressive CKD	Chronic heart failure
CRS type III Acute renocardiac syndrome	Primary acute kidney injury leading to cardiac dysfunction	AKI Volume overload Uremia Dyselectrolitemia
CRS type IV Chronic renocardiac syndrome	Primary chronic kidney disease leading to cardiac dysfunction	Chronic glomerular disease Hypertension Anemia Cronary arteries disease
CRS type V Secondary cardiorenal syndrome	Both cardiac and renal dysfunction caused by systemic condition	Diabetes mellitus Systemic infection Amyloidosis Vasculitis

CRS—cardiorenal syndrome, ADHF—acute decompensated heart failure, CKD—chronic kidney disease, AKI—acute kidney disease.

**Table 2:** Definitions of acute kidney injury.

Stage	KDIGO	AKIN	RIFLE	Urinary Output
1 [R]isk	sCr 1.5–1.9× baseline in 7 days / ≥0.3 mg/dL increase in 48 h	sCr 1.5–2× baseline in 7 days / ≥0.3 mg/dL increase in 48 h	sCr 1.5 baseline in 7 days / GFR decrease >25%	<0.5 mL/kg/h in 6 h
2 [I]njury	sCr 2–2.9× baseline	sCr 2–3× baseline	sCr 2× baseline / GFR decrease >50%	<0.5 mL/kg/h in 12 h
3 [F]ailure	sCr ≥ 3× baseline / sCr ≥ 4 mg/dL / Initation of RRT	sCr ≥ 3× baseline / sCr ≥ 4 mg/dL (with acute increase of ≥0.5 mg/dL) / Initiation of RRT	sCr ≥ 3× baseline / sCr ≥ 4 mg/dL (with acute increase of ≥0.5 mg/dL) / GFR decrease >75%	<0.3 mL/kg/h in 24 h / Anuria in 12 h
[L]oss			Loss of kidney function >4 weeks	
[E]nd stage renal disease			End-stage kidney disease >3 months	
**WRF**				
Equivalent to stage 1 according to AKIN or Risk according to RIFLE / Based on CysC increase >0.3 mg/dL				

KDIGO—Kidney Disease Improving Global Outcomes, AKIN—Acute Kidney Injury Network, RIFLE—risk, injury, failure, loss, end-stage kidney disease, sCr—serum creatinine, GFR—glomerular filtration rate, RRT—renal replacement therapy, WRF—worsening of renal function.

**Table 3:** Characteristics of tubular injury biomarkers.

Marker	Origin and Features	Strengths	Limitations
NGAL	Mainly produced by neutrophils; freely filtered through the glomerulus, reabsorbed by proximal tubule; involved in innate immunity	Early marker of AKI, strong prognostic of 30-day outcome and WRF in CRS I	May be confounded by hypertension, infections, hypoxemia, anemia and malignancies
KIM-1	Transmembrane glycoprotein, expressed by the proximal tubular cells in response to ischemia	Early marker of AKI, risk stratification of WRF in CRS I, sensitive and specific	Scarce data on prognostic value in CRS I, needs standarization
NAG	Lysosomal enzyme, produced by proximal tubules	Early marker of AKI, prognosis of mortality, worsening HF and rehospitalization	Inhibited by endogeneous urea, increased in autoimmune and inflammatory diseases, hyperthyroidism and impaired glucose tolerance
Interleukin-18	Proinflammatory cytokine produced by proximal tubules, activated in NLRP3 inflammasome	Risk stratification of WRF and death in CRS I	Increased in autoimmune and inflammatory diseases, metabolic syndrome, DM 2, sepsis
Urinary angiotensinogen	Produced by liver and proximal tubules, marker of RAAS activation	Early marker of AKI, strong predictor of the WRF and death in CRS I	May be confounded by using RAAS inhibitors; increased in hypertension and DM
Uromodulin	Glycoprotein produced by tubular cells of thick ascending limb and early distal tubules; regulates salt handling, involved in innate immunity	Risk stratification for cardiovascular events, death and progression of kidney disease	Large fluctuations depending on diet, hydration state and medications
TIMP-2	Cellular stress biomarkers, involved in cell cycle; secreted by renal tubules	Early marker of AKI in CRS I	Unclear prognostic value, may be increased in inflammation, ischemia and others
IGFBP7			
FABPs	Take part in fatty acid transport and metabolism, isolated in variety of tissues	Early marker of AKI in CRS I	Unclear prognostic value in CRS, not kidney-specific
B2M	Component of MHC class I, secreted by nucleated cells, reabsorbed by proximal tubules	Possibly an early indicator of AKI, linked to severity of CRS	Poor standardization of collection protocols, unstable in urine, limited data in HF, increased in chronic inflammatory conditions and infections
A1M	Protein synthesized by the liver; antioxidant effect; reabsorbed by the proximal renal tubule	Risk stratification for AKI, prognosis of kidney disease progression and all-cause mortality, independent from GFR and albuminuria	Elevated in presence of acute stressors, e.g., surgery

NGAL—neutrophil gelatinase-associated lipocalin, AKI—acute kidney injury, WRF—worsening of renal function, CRS—cardiorenal syndrome, KIM-1—kidney injury molecule-1, NAG—N-acetyl-β-D-glucosaminidase, HF—heart failure, DM—diabetes mellitus, RAAS—renin-angiotensin-aldosterone system, TIMP-2—tissue inhibitor of metalloproteinases 2, IGFBP-7—insulin-like growth factor binding protein 7, FABPs—fatty-acid-binding proteins, B2M—beta-2-microglobulin, A1M—alpha-1-microglobulin.

**Table 4:** Comparison of renal imaging modalities.

Modality	Features	Strengths	Limitations
Ultrasonography	Bidimensional morphological evaluation	Fast, cost effective, safe, feasible at the bedside, hemodynamic diagnostic and prognostic biomarkers	Low specificity, depends on operator
Doppler-enchanced ultrasonography	Arterial and venous hemodynamic evaluation		Changed by vascular alterations, susceptible to arrhythmias, depends on operator
Computed tomography	Morphological and functional evaluation	Availability, high resolution of images	The possibility of contrast-induced nephropathy, radiation exposure, not applicable for serial assessments
Magnetic resonance imaging	Morphological and functional evaluation	No radiation exposure, suitable for longitudinal assessment	Expensive, requires patient compliance, time consuming, gadolinum-based contrast associated with nephrogenic systemic fibrosis
DWI and DTI	Assessment of fibrotic changes	No contrast, reproducibility, possible prognostic value	No clinical use, needs further studies
ASL	Perfusion evaluation	No contrast, reproducibility, possible prognostic value	No clinical use, needs further studies
BOLD	Identification of hypoxia	No contrast	No clinical use, lack of standardization, needs further studies
Nuclear imaging	Functional evaluation	Low radiation dose	Poor image quality, radiation exposure
PET	Functional and metabolic evaluation	Low radiation dose, can be combined with CT/MRI	Expensive, time consuming, disturbed results in diabetes, not applicable for serial assessments, limited accessibility

DWI—diffusion-weighted imaging, DTI—diffusion tensor imaging, ASL—arterial spin labeling, BOLD—blood oxygen level dependent, PET—positron emission tomography, CT—computed tomography, MRI—magnetic resonance imaging.
